# Analysis of the chemotherapeutic effects of a propadiene compound on malignant ovarian cancer cells

**DOI:** 10.18632/oncotarget.11012

**Published:** 2016-08-02

**Authors:** Shuqing Li, Lina Yang, Jingshu Wang, Fan Liang, Bin Chang, Huafen Gu, Honglin Wang, Gong Yang, Yaping Chen

**Affiliations:** ^1^ Department of Obstetrics and Gynecology, The Fifth People's Hospital of Shanghai, Fudan University, Shanghai 200240, China; ^2^ Department of Pathology, Fudan University Shanghai Cancer Center, and Department of Oncology, Shanghai Medical College, Fudan University, Shanghai 200032, China; ^3^ Cancer Institute, Fudan University Shanghai Cancer Center, and Department of Oncology, Shanghai Medical College, Fudan University, Shanghai 200032, China; ^4^ Central laboratory, The Fifth People's Hospital of Shanghai, Fudan University, Shanghai 200240, China

**Keywords:** ovarian cancer, propadiene compound, PI3K/Akt, MAPK, ATM/Chk2

## Abstract

Epithelial ovarian cancer is most lethal in female reproductive carcinomas owing to the high chemoresistance and metastasis, so more efficient therapeutic agents are terribly needed. A propadiene compound: 1-phenylpropadienyl phosphine oxide (PHPO), was employed to test the chemotherapeutic efficacy against ovarian cancer cell lines. MTT assay showed that PHPO displayed a much lower IC50 than cisplatin and paclitaxel, while combination treatment of cells with PHPO + cisplatin induced more apoptosis than with PHPO + paclitaxel or with cisplatin + paclitaxel (*p* < 0.05). Animal assays demonstrated that subcutaneous tumor growth was highly inhibited by PHPO + cisplatin, compared with that inhibited by PHPO or by cisplatin treatment alone, indicating PHPO and cisplatin may have synergistic effects against ovarian cancer growth. We also found that PHPO induced few side effects on animals, compared with cisplatin. Mechanistic studies suggested that treatment of cells with PHPO or with PHPO + cisplatin differentially inhibited the PI3K/Akt, MAPK and ATM/Chk2 pathways, which consequently suppressed the anti-apoptotic factors Bcl-xL, Bcl-2 and XIAP, but activated the pro-apoptotic factors Bad, Bax, p53, caspase 9, caspase 8, caspase 7 and PARP. Taken together, PHPO may induce cell apoptosis through multiple signal pathways, especially when used along with cisplatin. Therefore, PHPO may be explored as a prospective agent to effectively treat ovarian cancer.

## INTRODUCTION

Epithelial ovarian cancer (EOC) accounts for only 4% of cancer deaths in women, but is most lethal in female reproductive carcinomas mainly due to the late diagnosis and peritoneal dissemination [1, 2]. The current standard therapies including radical surgery and platinum-based cytotoxic chemotherapy are initially effective [2, 3], but a significant number of patients may relapse and acquire chemoresistance to platinum-based chemotherapy, leading to shorter disease-free survivals [4]. Therefore, it is essential to develop novel therapeutic agents for the relapsing ovarian cancer patients, especially for those resistant to platinum-based cytotoxic agents.

The PI3K/Akt signaling is commonly activated and plays an important role in cancer cell proliferation, metastasis and chemoresistance [5, 6]. Amplification or mutations of PIK3CA (encoding the catalytic subunit of PI3K-p110α) and the phosphorylation of Akt can activate this pathway in roughly 70% of ovarian cancer [7]. In the PI3K/Akt pathway, PI3K initiates the survival mechanism through Akt that exerts anti-apoptotic effects, antagonizes cell cycle arrest and evokes chemoresistance via mTOR signaling [8, 9]. PI3Kα (PIK3CA) is a mostly studied subtype, whose mutations or abnormal expression are most commonly reported in different cancers [10]. In addition, the phosphorylated Akt activates the anti-apoptotic Bcl-2 family members (Bcl-xL, Bcl-2, Mcl-1, Bcl-w and A1), blocks the functions of the pro-apoptotic factors (Bak, Bad and Bax), and alters the activity of caspase-processing, leading to chemoresistance [8, 11]. Therefore, the targeted therapy against the PI3K/Akt signaling may be a promising strategy to efficiently treat cancers. XIAP is an anti-apoptotic protein in IAPs family that is characterized by the presence of a caspase recruitment domain and an NH_2_-terminal baculovirus inhibitor of apoptosis repeat motif. XIAP is a direct inhibitor of execution caspase 3 and caspase 7 and suppresses the Bax/cytochrome c pathway by inhibiting caspase 9[12, 13]. Furthermore, XIAP promotes cell survival though preserving the integrity and function of PI3K/Akt pathway [14].

Mitogen-activated protein kinases (MAPKs) are serine/threonine protein kinases that regulate a variety of cellular processes including proliferation and apoptosis [15]. Extracellular signal-regulated kinase (Erk) acts as a protective signaling pathway sustaining tumor cell survival and contributing to drug resistance. Erk inhibitors have been shown to successfully overcome drug resistance to some extent [16, 17]. Previous studies have shown that Jun N-terminal kinase (JNK) and p38 MAPK pathways display both positive and negative roles in modulating cell survival [18]. In normal and low passage proliferating cells, JNK and p38 MAPK pathways may initiate cellular transformation rather than execute apoptosis [19]. While in immortalized and cancer cells, they likely influence gene expressions and set the cellular susceptibility to apoptotic stimuli [20, 21]. Several studies have indicated that chemotherapeutic agents causes apoptosis through the activation of JNK and p38 pathways [21, 22].

ATM/Chk2 (Ataxia telangiectasia mutated kinase/the checkpoint kinase 2) is a major branch at the upstream of the DNA damage repair signaling. Chk2 is a stable protein being expressed throughout the cell cycle and largely inactive in the absence of DNA damage, but is activated mainly by ATM in response to double-strand DNA breaks (DSBs). Moreover, Chk2 phosphorylates the N-terminal activation domain of p53 and thereby regulates p53 in response to DSBs [23]. p53 blocks the functions of the anti-apoptotic Bcl-2 family members and activates some pro-apoptotic factors, while caspase 9, which initiates the executioner apoptotic caspase 3 and caspase 7, can be further activated by Bak or Bax [24]. Most of recurrent or metastatic cancer cells are featured with acquired chemoresistance due to the dysregulation of DNA repair networks associated with ATM/Chk2[25]. Rad51 plays a major role in homologous recombinational repair. Numerous studies report that Rad51 is over-expressed in different cancers, elevated expression of Rad51 is correlated with decreased patient survival. DNA damage is the main mechanism that cisplatin exerts antineoplastic effect as well as the major determinants of cisplatin resistance, which can be repaired by ATM/Chk2 signaling [26]. Therefore, inhibiting the ATM/Chk2 signaling enhances the sensitivity of cancer cells to cisplatin [27].

In this study, we investigated the primary function of PHPO in antitumor effect in terms of PI3K/Akt, MAPK and ATM/Chk2 pathways. We found PHPO, especially when used along with cisplatin, inhibited the PI3K/Akt, MAPK and ATM/Chk2 pathways, which consequently suppressed the anti-apoptotic factors Bcl-xL, Bcl-2 and XIAP, but activated the pro-apoptotic factors Bad, Bax, caspase 9, caspase 8, caspase 7 and PARP.

## RESULTS

### PHPO inhibits cell proliferation

**Figure 1:**
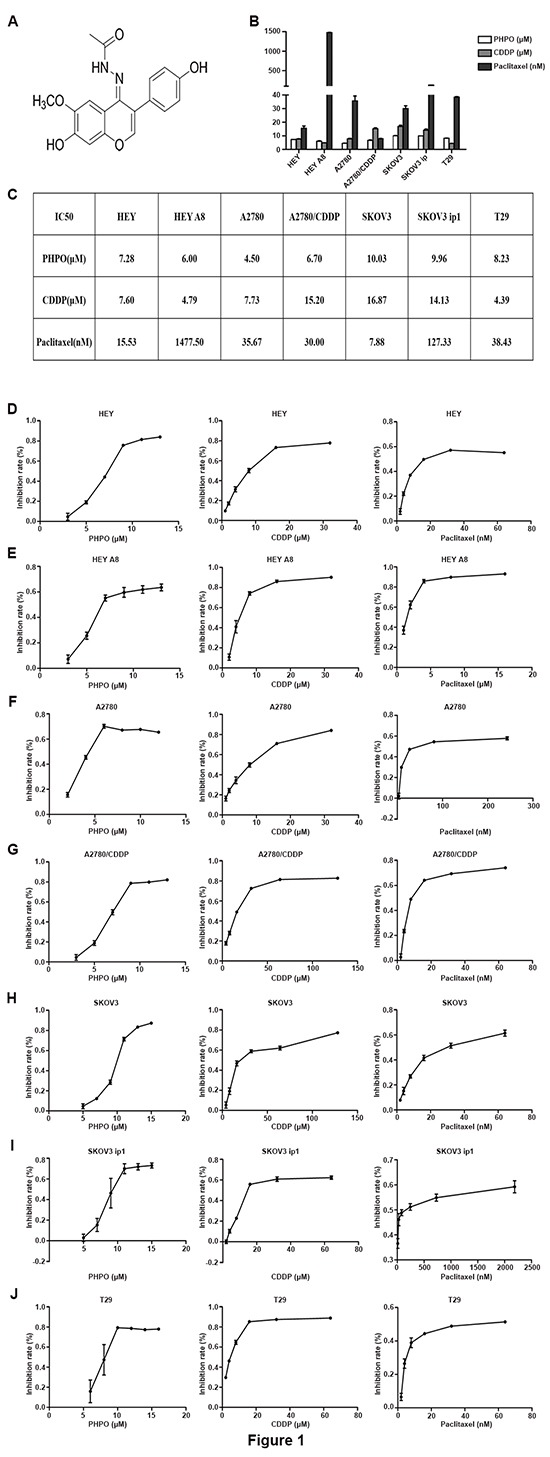
PHPO inhibits cell proliferation **A.** Chemical structure of 1-phenylpropadienyl phosphine oxide, PHPO. **B-C.** All IC50 values after treatment with various concentrations of PHPO, cisplatin or paclitaxel for 48 h. Data are mean ± SEM from three independent experiments. **D-J.** PHPO, cisplatin or paclitaxel inhibit the proliferation of HEY **(D)**, HEY A8 **(E)**, A2780 **(F)**, A2780/CDDP **(G)**, SKOV3 **(H)**, SKOV3 ip1 **(I)** and T29 **(J)** cell lines. Cell viability determined by MTT assay. The experiments were repeated three times, and representative experiments are shown.

The anti-proliferative activity of PHPO was tested by using normal human ovarian epithelial cells (T29) and human ovarian cancer cell lines including HEY, HEY A8, A2780, A2780/CDDP, SKOV3 and SKOV3 ip1. Cells were treated with different concentrations of PHPO, cisplatin or paclitaxel for 48 hours, and the cell viability was ascertained by MTT assay. The IC50 values were calculated and listed in Figure 1B and 1C. As shown in Figure 1D-1J, PHPO significantly inhibited the growth of all cell lines in a dose-dependent manner. On the contrary, the sensitivity of normal human ovarian epithelial cells (T29) to PHPO was much lower than human ovarian cancer cells, suggesting that PHPO may possess less cytotoxicity against normal ovarian epithelial cells. Moreover, compared with cisplatin, the cell growth inhibition by PHPO was more effective on human ovarian cancer cell lines HEY, A2780, A2780/CDDP, SKOV3 and SKOV3 ip1 (Figure 1D-1J).

**Figure 2:**
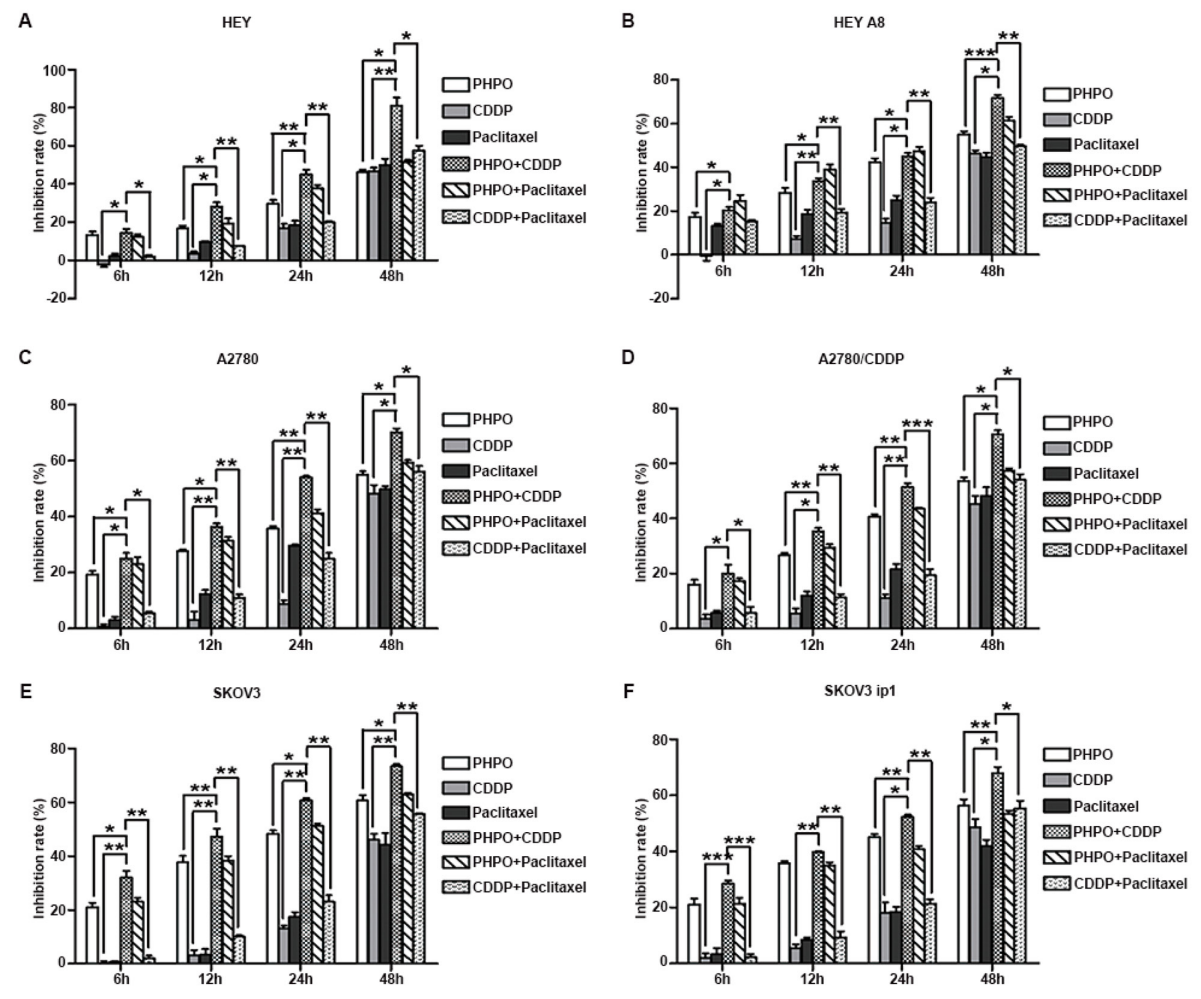
Anti-proliferative activity of combination treatment **A-F.** Growth inhibition rate of HEY **(A)**, HEY A8 **(B)**, A2780 **(C)**, A2780/CDDP **(D)**, SKOV3 **(E)**, SKOV3 ip1 **(F)** after treatment with PHPO, cisplatin, paclitaxel, PHPO + cisplatin, PHPO + paclitaxel or cisplatin + paclitaxel at the concentrations of IC50s for 6 h, 12 h, 24 h, 48 h. The experiments were repeated three times. Bars represent mean ± SEM, n=3. * refers to *p* < 0.05, ** refers to *p* < 0.01, *** refers to *p* < 0.001.

Next, we further tested the anti-proliferative activity of combination treatment by MTT assay. As shown in Figure 2A-2F, the inhibition of cell growth by single-agent treatments was much less than that by combination treatments (*p* < 0.05). The treatment of cells with PHPO + cisplatin induced more cell death than did the treatment with PHPO + paclitaxel or with cisplatin + paclitaxel (*p* < 0.05), the first line of chemotherapeutic agents for ovarian cancer, indicating the treatment of cells with PHPO + cisplatin was more effective. Based on these data, we performed the following experiments using PHPO, cisplatin or PHPO + cisplatin.

### PHPO inhibits the growth of xenograft ovarian tumor

**Figure 3:**
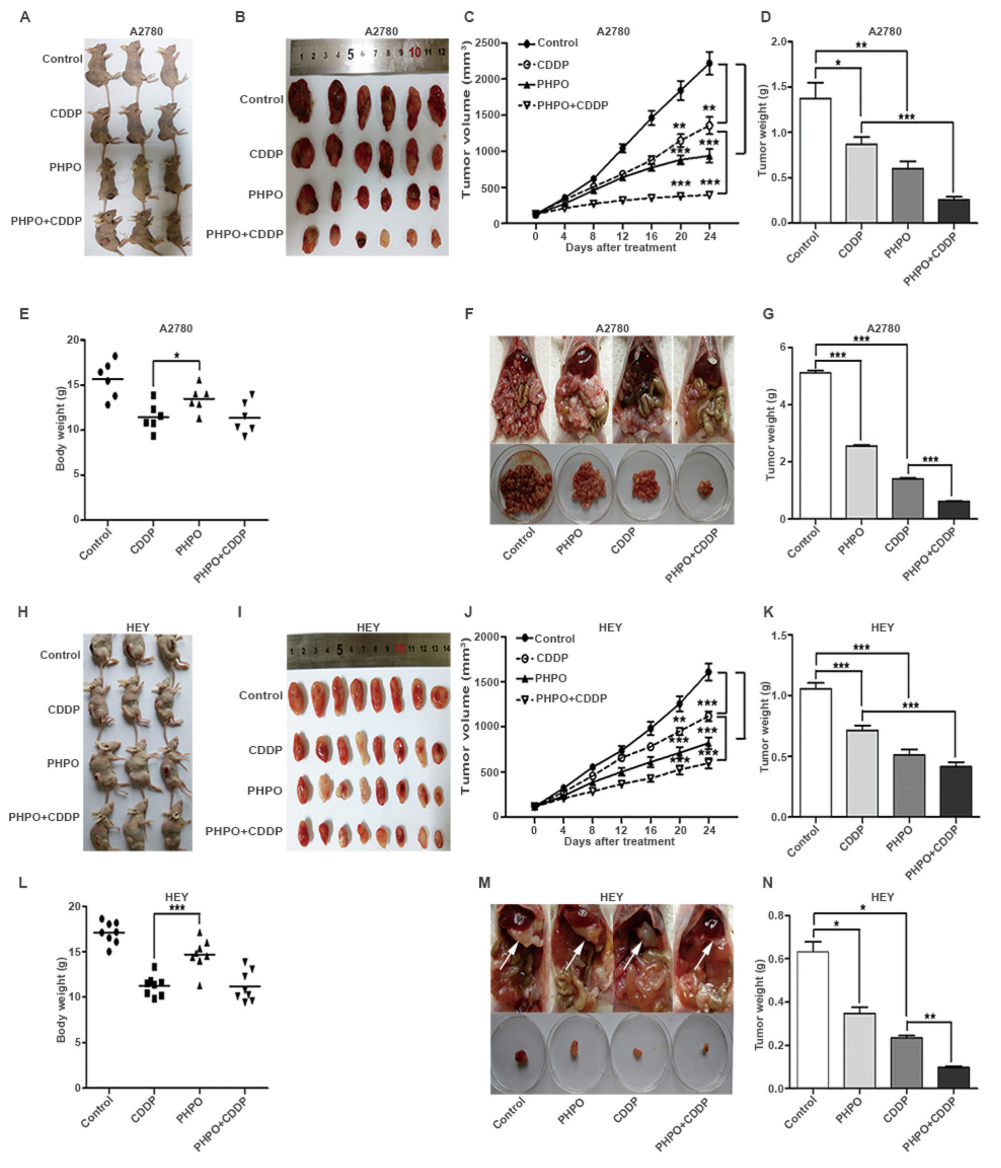
Inhibition of xenograft ovarian tumor by PHPO **A-E.** Mice were subcutaneously inoculated with A2780 and administrated with vehicle, PHPO (25mg/kg), cisplatin (5mg/kg) or PHPO (25mg/kg) + cisplatin (5mg/kg) (n = 6). Treatment was given though intraperitoneal injection every 4 days. Tumors removed **(A and B)**, the average tumor volumes **(C)**, weights **(D)** and body weights **(E)** are shown. **F-G.** Mice with intraperitoneal inoculation were treated as same as subcutaneous inoculation groups (n = 5). Tumor nodules removed **(F)** and the average tumor nodule weights **(D)** are shown. **H-L.** Mice were subcutaneously inoculated with HEY and administrated with the same treatment (n = 8). Tumors removed **(H and I)**, the average tumor volumes **(J)**, weights **(K)** and body weights **(L)** are shown. **M-N.** Mice with intraperitoneal inoculation were treated as same as subcutaneous inoculation groups (n = 3). Tumor nodules removed **(F)** and the average tumor nodule weights **(D)** are shown. Data are mean ± SEM, * refers to *p* < 0.05, ** refers to *p* < 0.01, *** refers to *p* < 0.001.

Based on the in vitro anti-proliferative activity of PHPO, we tested the in vivo anti-tumor activity in xenograft tumor models. Xenograft tumors were induced by subcutaneous inoculation in female BALB/C nude mice. When the tumor volume reached roughly 100mm^3^, mice were randomly divided into four groups and administrated with vehicle, PHPO (25mg/kg), cisplatin (5mg/kg) or PHPO (25mg/kg) + cisplatin (5mg/kg). In A2780 xenograft tumor models, compared with the vehicle-treated controls, treatments with cisplatin, PHPO and PHPO + cisplatin reduced tumor volumes by 39%, 58% and 82%, respectively (Figure 3A-3D). In HEY xenograft tumor models, the same treatments decreased tumor volumes by 32%, 56% and 69%, respectively (Figure 3H-3K). Compared with cisplatin or PHPO alone, treatment with PHPO + cisplatin mostly reduced the tumor volumes (*p* < 0.05). The body weights of mice treated with either cisplatin or PHPO + cisplatin were significantly decreased compared with that treated with PHPO (*p* < 0.05), indicating that PHPO may be less toxic than cisplatin (Figure 3E and 3L).

### PHPO suppresses the metastasis of xenograft ovarian tumor

To investigate the anti-metastatic efficacy of PHPO, we also established xenograft tumor models though intraperitoneal inoculation of A2780 or HEY cells in female BALB/C nude mice. Two weeks post the inoculation, mice were randomly divided into four groups and administrated with the same way for subcutaneously inoculated mice. At the end of experiment, mice were euthanized, and the tumor nodules were immediately removed and weighed. In A2780 xenograft tumor models, compared with the vehicle-treated controls, treatments with PHPO, cisplatin and PHPO + cisplatin reduced tumor nodule weights by 50%, 73% and 88%, respectively (Figure 3F and 3G). In HEY xenograft tumor models, the same treatments decreased tumor nodule weights by 45%, 63% and 85%, respectively compared with controls (Figure 3M and 3N). Treatment with PHPO + cisplatin significantly reduced tumor nodule weights compared with PHPO or cisplatin treatment (*p* < 0.05).

### Effect of PHPO and cisplatin on liver and kidney histopathology

**Figure 4:**
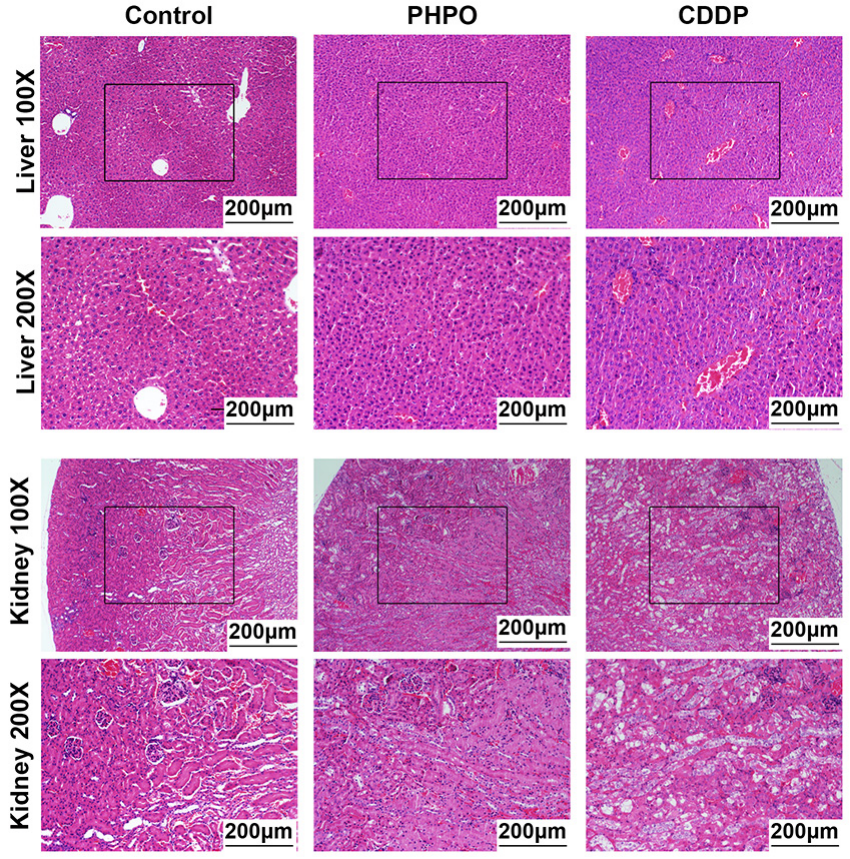
Representative images of liver and kidney stained with H&E staining Histological images of liver at 100 × magnification or 200 × magnification **(upper panels)**. Histological images of kidney at 100 × magnification or 200 × magnification **(lower panels)**.

Histological analysis of the vehicle-treated mice showed normal liver and kidney architecture. Mice treated with PHPO exhibited almost similar patterns. However, hepatic sections from cisplatin group exhibited disordered architecture with inflammatory cell infiltration, cytoplasmic degeneration, nuclear enlargement and disorganization (Figure 4 upper panels). Hematoxylin and eosin (H&E) staining of renal tissues from cisplatin group showed some marked histological changes such as infiltration of inflammatory cell, glomerular damage with detachment from basement membrane and tubular dilatation as compared to normal tissues possessing intact glomerular and tubular structures (Figure 4 lower panels).

### PHPO induces cell apoptosis

**Figure 5:**
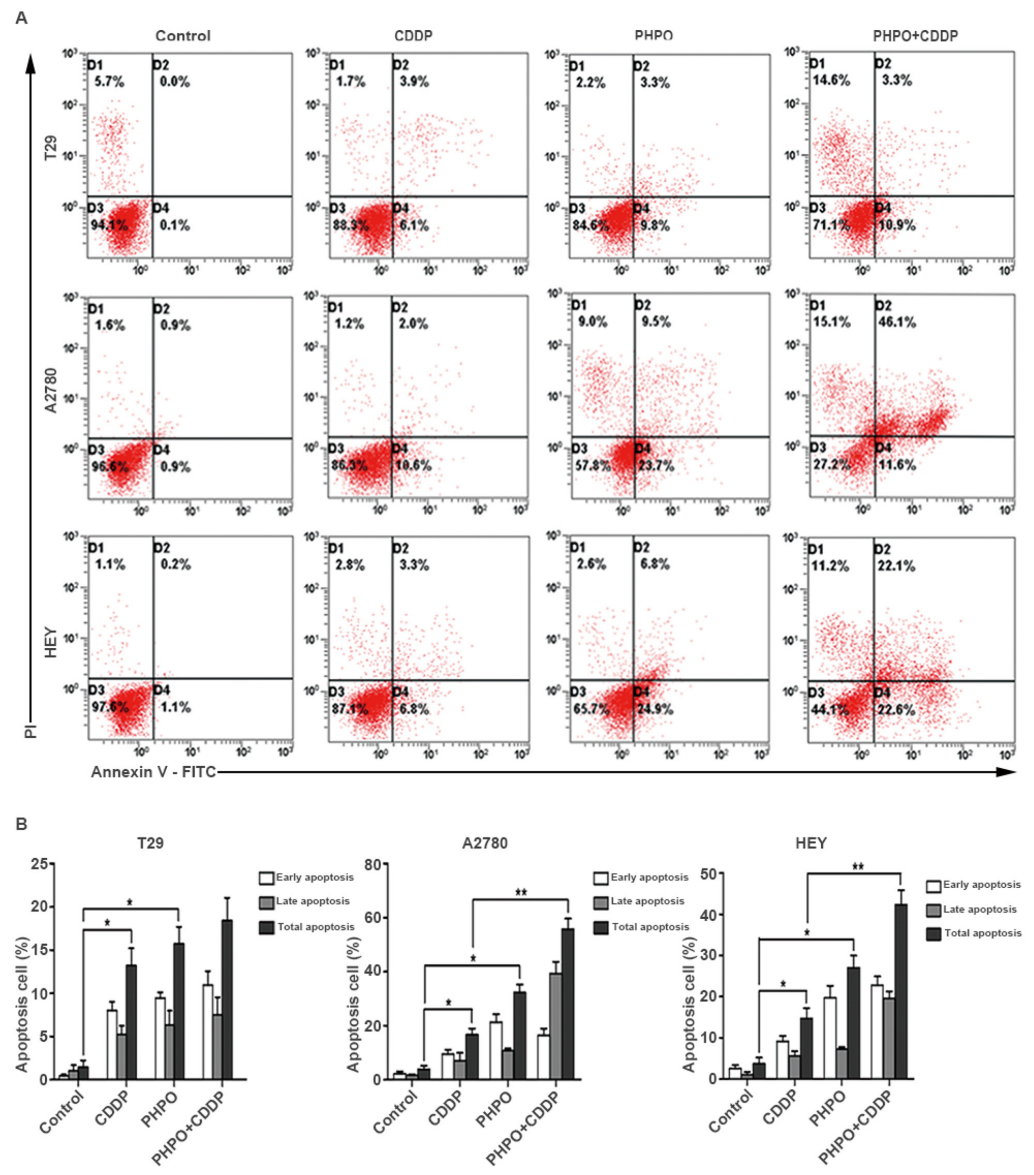
PHPO induces cell apoptosis **A.** Representative flow cytometry profiles of apoptosis. **B.** Quantitative results obtained using Annexin V/PI staining. Bars represent mean ± SEM, n=3. * refers to *p* < 0.05, ** refers to *p* < 0.01.

To determine whether PHPO induces apoptosis, three cell lines T29, A2780 and HEY were selected and treated with DMSO (diluent), PHPO, cisplatin or PHPO + cisplatin at the concentrations of IC50s for 24 h, then stained with Annexin-V and PI. The total proportions of early apoptosis and late apoptosis induced by PHPO + cisplatin were higher than that induced by either PHPO or cisplatin alone in A2780 and HEY cell lines (*p* < 0.05) (Figure 5A and 5B), while no changes were found between PHPO + cisplatin and single agent in T29 cells. These results clearly showed that the treatment of cells with PHPO + cisplatin evoked more apoptosis than did with PHPO or cisplatin alone.

**Figure 6:**
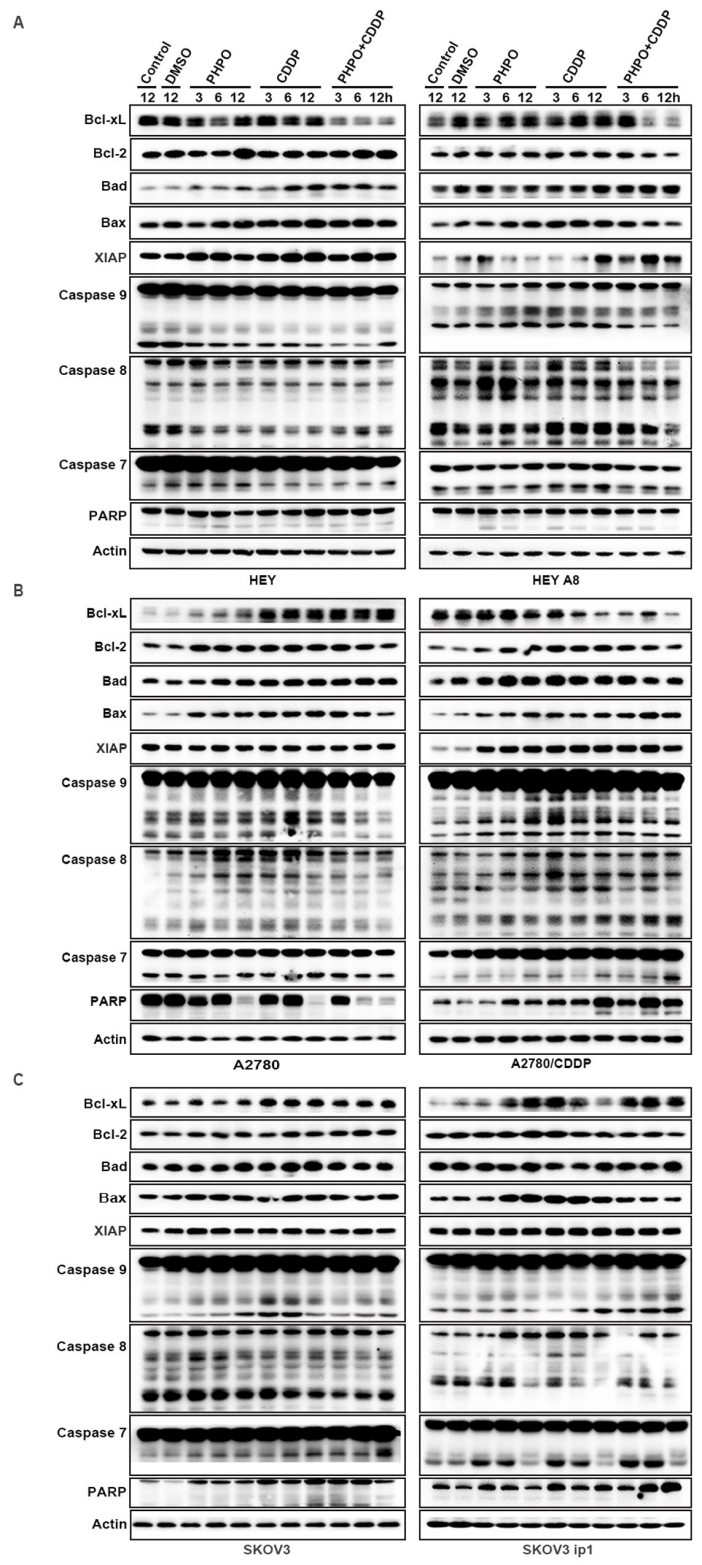
The mechanism of apoptosis induced by PHPO PHPO + cisplatin inhibited the anti-apoptotic factors (Bcl-xL, Bcl-2, XIAP) and activated the pro-apoptotic factors (Bad, Bax, caspase 9, caspase 8, caspase 7 and PARP) in **A.** HEY and HEY A8 **B.** A2780 and A2780/CDDP **C.** SKOV3 and SKOV3 ip1 cell lines.

Furthermore, we investigated the expression of some anti-apoptotic factors and pro-apoptotic factors by immunoblotting. In HEY cells, Bcl-xL was markedly inhibited by PHPO + cisplatin, while no changes were conceived by single agent treatment. The expression of Bcl-2 was enhanced by PHPO or PHPO + cisplatin treatment. Bad and Bax were time-dependently upregulated in All Drug Treatments (ADTs) compared with controls, while PHPO+ cisplatin induced the highest expression of Bad and Bax, followed by cisplatin. The level of anti-apoptotic factor XIAP was clearly increased in ADTs. The cleaved caspase 9 was decreased in ADTs, but the cleaved caspase 7 was suppressed only by cisplatin or PHPO + cisplatin. No clear changes of the cleaved caspase 8 were found, while the cleaved PARP was increased in ADTs. In HEY A8 cells, Bcl-xL and Bcl-2 were decreased by PHPO + cisplatin, but Bax and Bad were increased in ADTs. XIAP was promoted, especially in PHPO + cisplatin treated cells. The cleavage of caspase 9, caspase 8 and PARP was increased in ADTs. No remarkable changes of the cleaved caspase 7 were found in ADTs (Figure 6A).

In A2780 cells, both the anti-apoptotic factors Bcl-xL and Bcl-2 and pro-apoptotic factors Bad and Bax were increased. No changes of XIAP were found in ADTs. The cleaved caspase 9, caspase 8, caspase 7 and PARP were inhibited by PHPO + cisplatin treatment. In A2780/CDDP cells, Bcl-xL was gradually reduced in ADTs, but Bcl-2, XIAP, Bad, Bax and the cleaved caspase 9, caspase 8, caspase 7 and PARP were increased in ADTs compared with controls (Figure 6B).

In SKOV3 cells, Bcl-xL, Bcl-2, Bad, Bax and the cleaved caspase 9 were enhanced in ADTs. The cleavage of caspase 7 and PARP was increased in ADTs, especially in PHPO + cisplatin treated cells. In SKOV3 ip1 cells, PHPO or PHPO + cisplatin gradually enhanced the expression of Bcl-xL, while cisplatin promoted the expression of Bcl-xL only at 3 h. PHPO promoted Bcl-2 at 12 h and cisplatin at 3 h, while PHPO + cisplatin inhibited Bcl-2. Bad was equally increased at 12 h in ADTs. Bax was increased merely in PHPO and cisplatin treatments, but not in PHPO + cisplatin. The cleavage of caspase 9, caspase 7 and PARP was increased in ADTs, especially in PHPO + cisplatin treatment (Figure 6C). These results suggest that cell apoptosis may be regulated differentially by PHPO, cisplatin or PHPO + cisplatin in cancer cell lines with diffferent genetic background.

### PHPO inhibits the PI3K/Akt signaling

**Figure 7:**
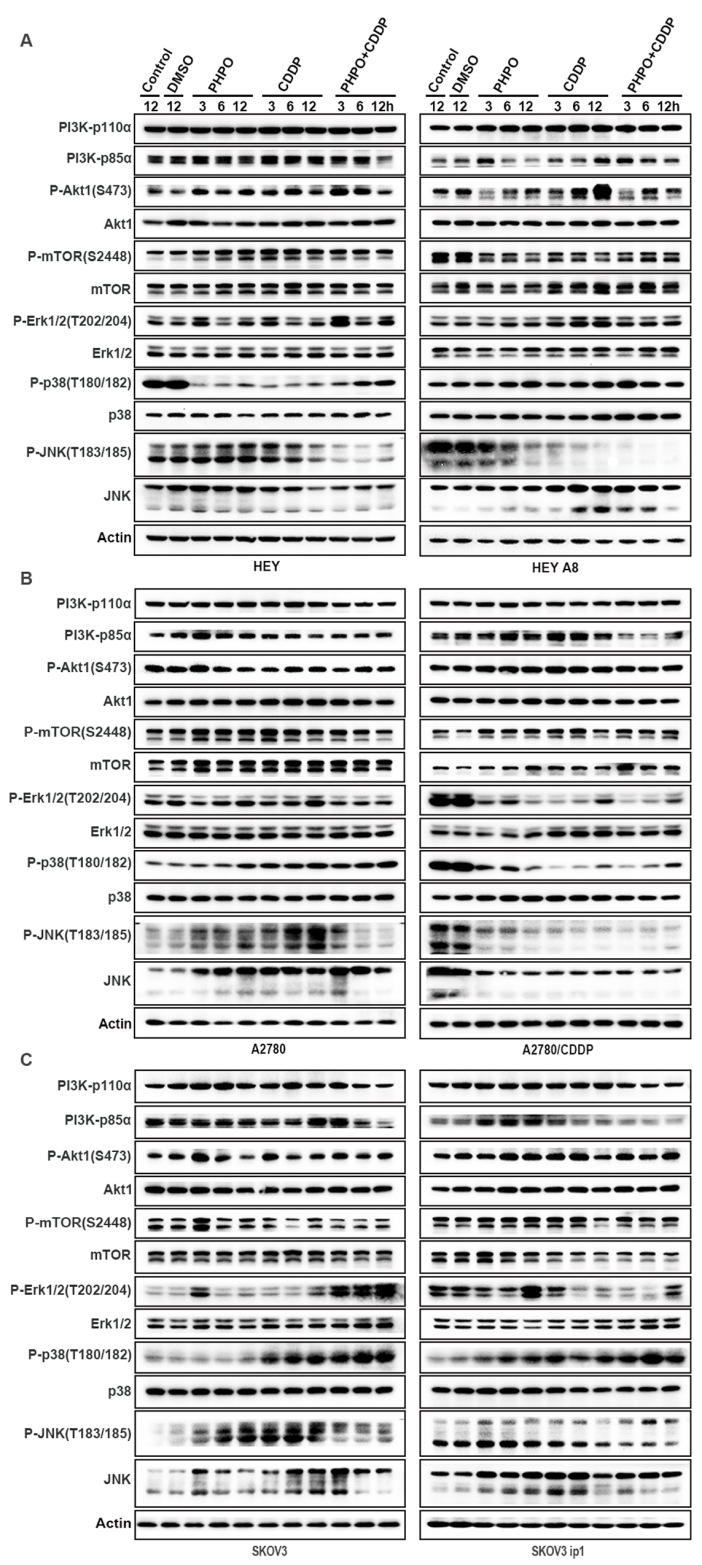
PHPO inhibits PI3K/Akt signaling and interrupts MAPK pathways Western blotting of PI3K/Akt and MAPK pathways in **A.** HEY and HEY A8 **B.** A2780 and A2780/CDDP **C.** SKOV3 and SKOV3 ip1 cell lines.

Activation of the PI3K/Akt signaling is associated with carcinogenesis and chemoresistance [28–30]. Therefore, Western blotting was employed to determine whether this pathway was involved in the PHPO + cisplatin-mediated anti-cancer efficacy. In HEY cells, PHPO and cisplatin increased the phosphorylation of Akt1 and mTOR. However, PHPO + cisplatin gradually suppressed the levels of PI3K-p85α, P-Akt1 and P-mTOR in a time-dependent manner. In HEY A8 cells, PHPO and PHPO + cisplatin downregulated the levels of PI3K-p85α and P-Akt1, while cisplatin promoted the expression of PI3K-p85α and P-Akt1. P-mTOR was inhibited by ADTs. The inhibition of cells with PHPO alone seemed more efficient than cisplatin or PHPO + cisplatin, indicating PHPO and cisplatin may have antistatic function in HEY A8 cells (Figure 7A). In A2780, SKOV3 and SKOV3 ip1 cells, PI3K-p110α, PI3K-p85α, P-Akt1 and P-mTOR were time-dependently reduced by PHPO + cisplatin compared with by PHPO or cisplatin alone. In A2780/CDDP cells, PI3K-p110α, P-Akt1 and P-mTOR were not inhibited by ADTs despite PI3K-p85α was decreased by PHPO + cisplatin. No clearly changes in total Akt1 and mTOR were found in all cell lines except in SKOV3 ip1 (Figure 7B and 7C). HEY A8 is more aggressive than HEY. PHPO and cisplatin seemed antistatic in HEY A8 cells, but PHPO+cisplatin still succeeded in reducing the levels of PI3K-p85α and P-Akt1. A2780/CDDP is resistant to cisplatin, only PHPO + cisplatin inhibited PI3K-p85α. SKOV3 ip1 is resistant to paclitaxel, PHPO + cisplatin time-dependently suppressed the expression of PI3K-p110α, PI3K-p85α, P-Akt1 and P-mTOR. These results demonstrate that PHPO + cisplatin could successfully inhibit the PI3K/Akt signaling to induce apoptosis in chemoresistant or aggressive ovarian cancer cell lines.

### PHPO interrupts the MAPK signaling

The MAPK pathways regulate the activity of genes involved in cell proliferation, DNA damage repair, cell cycle progression and apoptosis [31]. Three major MAPK pathways have been identified: the Erk family or classical pathway, c-jun N-terminal kinase (JNK), p38 MAPK [17, 32].

In HEY cells, ADTs clearly stimulated the phosphorylation of Erk1/2 at 3 h and 12 h, while PHPO + cisplatin induced the highest expression of these proteins. The phosphorylation of p38 was apparently suppressed in ADTs, but displayed an ascending trend as time went on (especially in PHPO + cisplatin treatment) presumably due to the decreased drug concentration. No changes of P-SAPK/JNK and JNK were found in PHPO treatment. In cisplatin and PHPO+ cisplatin treatments, P-SAPK/JNK and JNK were decreased significantly. In HEY A8 cells, the phosphorylation of Erk1/2 was slightly enhanced in ADTs. In all drug treated cells (especially in PHPO + cisplatin treatment), P-p38 was clearly increased while the level of P-SAPK/JNK was markedly decreased in a time-dependent manner compared with in control cells. But the total JNK was enhanced in cisplatin and PHPO + cisplatin treated cells (Figure 7A).

In A2780 cells, P-Erk1/2 was time-dependently increased by PHPO and cisplatin alone, but was inhibited by PHPO + cisplatin treatment. P-p38 in ADTs, especially in PHPO + cisplatin treated cells, was increased in a time-dependent manner. P-JNK was promoted by PHPO or cisplatin treatment, but was suppressed by PHPO + cisplatin treatment after 3 h. The total JNK was improved by ADTs. In A2780/CDDP cells, P-Erk1/2, P-p38 and P-JNK and the total JNK were decreased (Figure 7B).

In SKOV3 cells treated with PHPO + cisplatin, P-Erk1/2 was markedly enhanced compared with cells treated with PHPO or cisplatin alone. P-p38 was increased in cisplatin or PHPO + cisplatin treated SKOV3 cells, but not in PHPO treated cells. PHPO and cisplatin treatment improved P-SAPK/JNK, but PHPO + cisplatin treatment had the opposite effect, while the total JNK was increased in SKOV3 cells. In SKOV3 ip1 cells, P-Erk1/2 was promoted by PHPO at 12 h, but was suppressed by cisplatin or PHPO + cisplatin in different time. The levels of P-p38 and P-SAPK/JNK were increased in a time-dependent manner in ADTs (especially in PHPO + cisplatin treatment). The total level of JNK was improved (Figure 7C). These results suggest that PHPO, especially when used along with cisplatin, could successfully interrupt MAPK pathways in ovarian cancer cells.

### PHPO suppresses the ATM/Chk2 pathway

**Figure 8:**
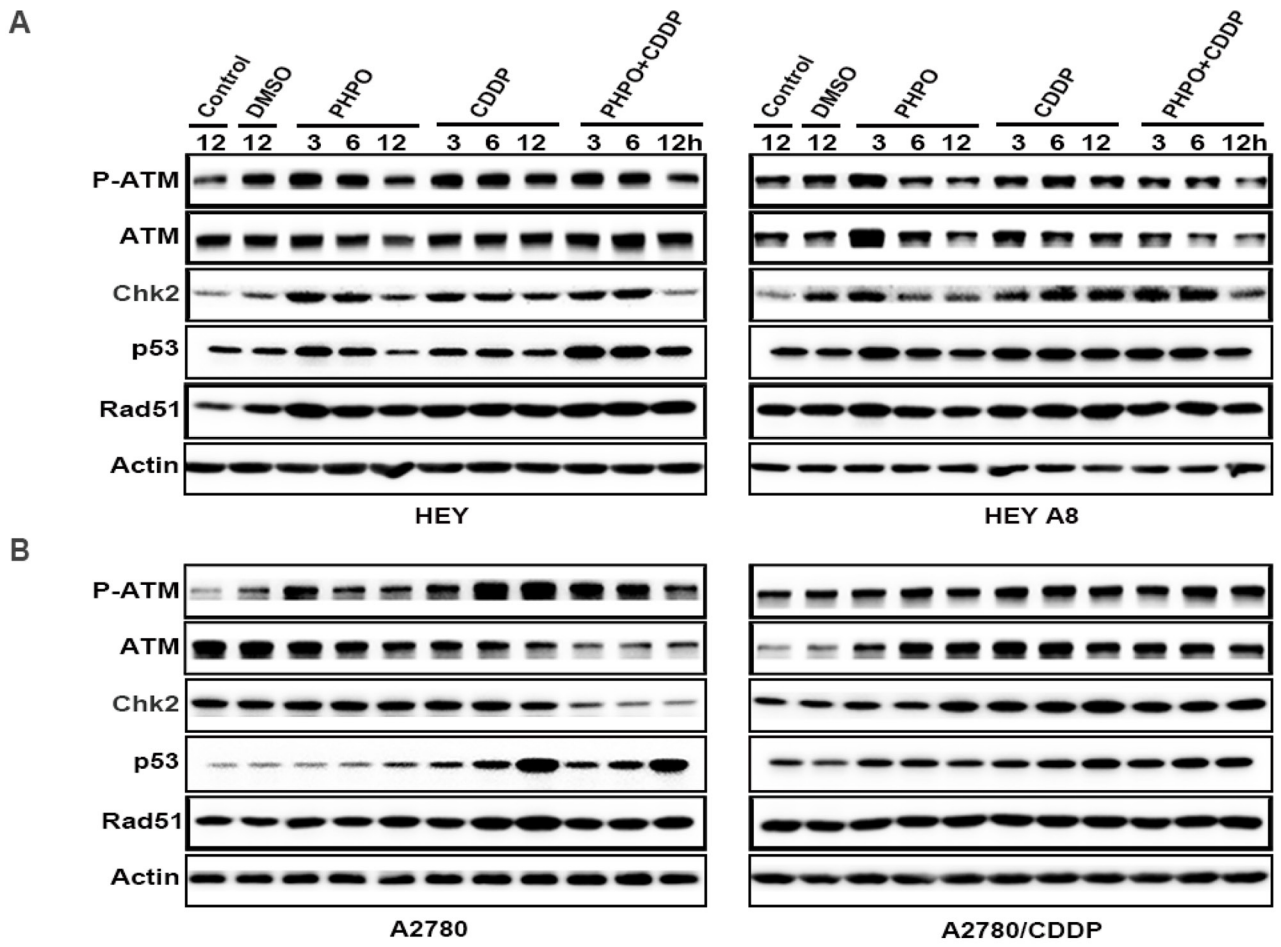
PHPO suppresses the ATM/Chk2 pathway The expression of ATM/Chk2 pathway in **A.** HEY and HEY A8 **B.** A2780 and A2780/CDDP cell lines.

Irreparable DNA damage is the essential mechanism to induce cancer cells apoptosis for chemotherapeutic compounds. Because ATM/Chk2 functions at the upstream of the DNA repair signaling, we detected the expression of ATM/Chk2 pathway by Western blotting. In HEY cells, the phosphorylation of ATM was time-dependently suppressed in ADTs. ATM was decreased in PHPO treated cells, no changes were found in cisplatin treatment, but ATM was enhanced in PHPO + cisplatin treated cells. ADTs inhibited the expression of Chk2 and p53 in a time-dependent manner. Rad51 was promoted in ADTs. In HEY A8 cells, the levels of P-ATM, ATM, Chk2, p53 and Rad51 were decreased in a time-dependent manner in PHPO and PHPO + cisplatin treatments. In cisplatin treatment, P-ATM, ATM, Chk2, p53 and Rad51 were improved (Figure 8A). In A2780 cells, P-ATM was apparently increased and ATM was time-dependently inhibited in ADTs. Chk2 was suppressed by PHPO + cisplatin treatment, but was improved by cisplatin treatment. p53 and Rad51 were promoted in a time-dependent manner in ADTs, especially in cisplatin treatment. In A2780/CDDP cells, P-ATM, ATM, Chk2 and p53 were enhanced in ADTs. No changes of Rad51 were found in ADTs (Figure 8B). These results indicate that PHPO could inhibit the ATM/Chk2 pathway to block DNA damage repair and enhance the sensitivity of cancer cells to cisplatin.

## DISCUSSION

Epithelial ovarian cancer is most lethal in female reproductive carcinomas [1]. Radical surgery and platinum-based chemotherapy is the current standard therapies for advanced ovarian cancer patients [2, 33]. Cisplatin and paclitaxel are the most commonly used compounds in clinic [34, 35]. The high mortality is partially due to the fact that many advanced cancer patients become refractory to current chemotherapeutic agents, leading to tumor recurrence and death [36]. Therefore, more efficient therapeutic agents are urgently needed. In this study, we investigated the treatment efficacy of PHPO and the combination of PHPO and cisplatin in chemoresistant or aggressive ovarian cancer cell lines. Our data demonstrate that PHPO may be a profound agent to effectively treat ovarian cancer.

Our data showed that the inhibition of cell proliferation by single agent is less effective than that by combination treatments. Treatment of cells with PHPO + cisplatin induced more cell death than did with PHPO + paclitaxel or with cisplatin + paclitaxel, while immortalized normal cells treated with PHPO displayed less sensitivity to apoptosis compared with cancer cells. It is well known that the platinum + paclitaxel chemotherapy has been the first-line treatment for ovarian cancer patients [37]. However, development of chemoresistance increasingly becomes a major impediment to successful treatment [38, 39]. In addition, most of patients suffering from ovarian cancer could not tolerate the traditional chemotherapy because of the drug toxicity [40]. Given these aspects, many studies have been focused on discovery of novel effective agents with little toxicity [41, 42]. Since platinum combination treatment was superior to single-agent platinum, and platinum-containing regimens were superior to the same regimen without platinum [42], which was consistent with our results, we attempted to utilize PHPO + cisplatin as a new strategy to treat ovarian cancer.

The in vivo anti-tumor assays demonstrated that, compared with cisplatin or PHPO treatment alone, treatment with PHPO + cisplatin significantly reduced subcutaneous tumor volumes (*p* < 0.05). The body weights of mice treated with cisplatin or PHPO + cisplatin were significantly decreased compared with PHPO treatment (*p* < 0.05), indicating that PHPO may be less toxic than cisplatin (Figure 3E and 3L), which was also evidenced by histological analysis of liver and kidney tissues (Figure 4). In intraperitoneal xenograft tumor models, treatment with PHPO + cisplatin significantly reduced tumor nodule weights compared with PHPO or cisplatin treatment (*p* < 0.05). These results indicate that the combination treatment of ovarian cancer cells or animals burdened with tumors using PHPO + cisplatin potentially inhibits ovarian cancer cell proliferation and metastasis.

To gain insight into the mechanism of apoptosis induced by PHPO+ cisplatin, immunoblotting was performed to examine the expression of PI3K/Akt, MAPK, ATM/Chk2 and apoptosis-associated proteins. The results showed that PHPO+ cisplatin suppressed the anti-apoptotic factors Bcl-xL, Bcl-2 and XIAP, but activated the pro-apoptotic factors Bad, Bax, caspase 9, caspase 8, caspase 7 and PARP though inhibiting the PI3K/Akt, MAPK, ATM/Chk2 pathways in both chemosensitive and chemoresistant ovarian cancer cell lines. However, whether PHPO has a unique and specific function in terms of these or unknown signal pathways still needs further investigation.

In conclusion, our primary data suggest that PHPO is a powerful agent to induce apoptosis through multiple signal pathways, especially when used along with cisplatin in chemoresistant or aggressive ovarian cancer cell lines. Thus the combination of PHPO and cisplatin may be a prospective agent to highly increase the treatment efficacy of ovarian cancer.

## MATERIALS AND METHODS

### Drugs

1-phenylpropadienyl phosphine oxide, named as PHPO (Figure 1A), is a propadiene compound, it was synthesized as follows:


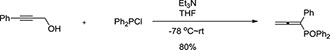


The reaction of 3-phenylprop-2-yn-1-ol (1.445g, 11.1mmol), Et3N (2.3mL, d = 0.726g/mL, 1.67g, 16.5mmol), and Ph2PCl (3.640g, 16.5mmol) in THF (100mL) afforded 2.778g (80%) of 1p: solid, mp127-129 °C (ethyl acetate / petroleum ether).

### Cell culture

Human ovarian cancer cell lines HEY, HEY A8, A2780, SKOV3 and SKOV3 ip1[43] were purchased from ATCC. A2780/CDDP and an immortalized normal human ovarian epithelial cell line (T29) were maintained in the laboratory of cancer Institute, Fudan University Shanghai Cancer Center. HEY A8, A2780/CDDP and SKOV3 ip1 derived from HEY, A2780 and SKOV3, respectively, are resistant to cisplatin or paclitaxel [44, 45]. Cells were routinely cultured with RPMI 1640 supplemented with 10% fetal bovine serum (FBS), antibiotics (100U/mL penicillin and 100μg/mL streptomycin) in a humidified incubator at 37°C and 5% CO_2_. Both RPMI 1640 medium and fetal bovine serum (FBS) were purchased from Thermo Scientific.

### MTT cytotoxicity assay

The in vitro cytotoxicity of PHPO, cisplatin and paclitaxel was determined by standard procedure of MTT (Sigma-Aldrich) assay [46]. Briefly, cells were seeded in 96-well plates at a density of 5000 cells/well. After overnight incubation, they were treated with a range of different concentrations of PHPO, cisplatin or paclitaxel and incubated at 37°C for 48 h. Then, the medium was replaced with 180μL of fresh medium along with 20μL of MTT solution (MTT dissolved in PBS at 5mg/mL) in each well and incubated at 37°C for 4 h. Finally, the MTT - containing medium was discarded and 150μL of DMSO per well was added to dissolve the newly formed formazan crystals. Absorbance of each well was determined by a microplate reader (Synergy H4, BioTek) at a 490nm wavelength. Growth inhibition rates were calculated with the following equation: Inhibition ratio = (OD_DMSO_ - OD_drug_)/ (OD_DMSO_ - OD_blank_) × 100%. Following this, the IC50s of the three drugs in six human ovarian cancer cell lines were utilized to perform synergistic effect assay. Specifically, the six human ovarian cancer cell lines were treated with DMSO (diluent), PHPO, cisplatin, paclitaxel, PHPO+ cisplatin, PHPO + paclitaxel or cisplatin + paclitaxel for 6 h, 12 h, 24 h, 48 h separately. And the subsequent procedures were described as above.

### In vivo tumor growth assay

All animal studies were approved by Animal Care and Use Committee of Fudan University Shanghai Cancer Center. Female BALB/c nude mice (4-5 week-old) were purchased from Shanghai Slac Laboratory Animal Co. Ltd. and housed in a specific pathogen free facility. Mice were subcutaneously inoculated with 5 × 10^6^ A2780 or HEY cells or intraperitoneally inoculated with 1 × 10^7^A2780 or HEY cells. After tumors reached 100mm^3^ in average, mice with subcutaneous inoculation were randomly divided into four groups (n = 6 for A2780 cells, n = 8 for HEY cells) and were treated with vehicle control (0.1mL DMSO and olive oil), PHPO (25mg/kg, compound dissolved in 0.1mL DMSO and olive oil), cisplatin (5mg/kg) or PHPO (25mg/kg, compound dissolved in 0.1mL DMSO and olive oil) + cisplatin (5mg/kg) though intraperitoneal injection. Administration of vehicle or agents and measurement of tumor volumes as well as body weight of each mouse were done once every 4 days for 3 weeks. Tumor volumes were calculated by the following formula: V = L × W^2^ × 0.52, where V is the volume, L is the length, and W is the width. At the same time, mice with intraperitoneal inoculation were also randomly divided into four groups (n = 5 for A2780 cells, n = 3 for HEY cells) and were treated as same as subcutaneous inoculation groups. At the end of experiment, all mice were euthanized, and the tumor nodules were immediately removed and weighed. The animal studies were conducted with the approval of the Animal Ethics Committee of Fudan University.

### Histopathology analysis

The tissue samples of liver and kidney were prepared from euthanized mice with tumor xenografts as previously described. The formalin-fixed and paraffin-embedded tissues were sliced into 3-μm-thick sections and stained with hematoxylin and eosin (H&E). All images were observed and photographed at 100 × or 200 × magnification.

### Cell apoptosis analysis

Cell apoptosis was detected with the Annexin V-fluorescein isothiocyanate (FITC)/ propidium iodide (PI) apoptosis assay kit (BD Biosciences). Cell lines T29, A2780 and HEY were incubated with DMSO (diluent), PHPO, cisplatin or PHPO + cisplatin at the concentrations of IC50s for 24 h. The cells were harvested, washed twice with cold 1 × PBS and resuspended in 500μL binding buffer at a density of 1 × 10^5^cells/mL. The cells were then stained with 5μL Annexin V-FITC and 5μL PI for 15min in dark condition at room temperature and subjected to analysis by flow cytometry (Cytomics FC 500MPL, Beckman Coulter). The early apoptosis was evaluated based on the percentage of cells with Annexin V + / PI - (the right lower quadrant, representing early apoptosis), while the late apoptosis was that of cells with Annexin V + / PI + (the right upper quadrant, representing late apoptosis). The results were indicated as mean values from three independent determinations.

### Western blotting analysis

Western blotting was performed to determine the expression levels of various apoptosis-related proteins in cells treated with DMSO (diluent), PHPO or PHPO + cisplatin at the concentrations of IC50s for 3 h, 6 h, 12 h. Cells were harvested, washed with cold 1 × PBS and lysed with RIPA lysis buffer (Beyotime) for 30min on ice, then centrifuged at 10, 000g for 15min at 4°C. The total protein concentration was determined by BCA protein assay kit (Beyotime). Equal amounts of protein samples were subjected to SDS-PAGE electrophoresis and transferred on to polyvinylidene fluoride (PVDF) membranes (Millipore). The membranes were blocked in 5% non-fat milk powder (2 h, room temperature), then incubated with primary antibodies (2 h, room temperature), and subsequently with secondary antibodies (1 h, room temperature) conjugated with horseradish peroxidase (HRP). Protein bands were developed with the chemiluminescent reagents (Millipore). Antibodies against XIAP, caspase 7, caspase 8, caspase 9, PARP, P-Erk1/2 (Thr202/Tyr204), Erk1/2, P-p38 (Thr180/Tyr182), p38, P-SAPK/JNK (Thr183/Tyr185), SAPK/JNK, P-Akt1 (Ser473), Akt1, P-mTOR (Ser2448), mTOR, P-ATM (Ser1981) and ATM were from Cell Signaling Technology. Antibodies against Bcl-xL, Bcl-2, Bad, Bax, PI3K-p110α, PI3K-p85α, Chk2, p53 and Rad51 were from Santa Cruz Biotechnology. The antibody against Actin was supplied by Sigma-Aldrich.

### Statistical analysis

GraphPad Prism was used for statistical analysis. All data were expressed as mean ± SEM (standard error of mean). The values of IC50 were shown by using a nonlinear regression model with a sigmoidal dose response. Comparisons between controls and treated groups involved paired t test or one-way ANOVA followed by Turkey's multiple comparison tests. A *p* value < 0.05 is considered statistically significant (* refers to *p* < 0.05, ** refers to *p* < 0.01, *** refers to *p* < 0.001). Combination index was calculated by CompuSyn software discovered by Chou T.C.et al.
